# Nephrotoxicities

**DOI:** 10.12688/f1000research.10192.1

**Published:** 2017-01-19

**Authors:** Stuart L. Goldstein

**Affiliations:** 1 Center for Acute Care Nephrology, Cincinnati Children’s Hospital Medical Center, Cininnati, OH, USA

**Keywords:** acute kidney injury, AKI, NINJA, nephrotoxic injury, nephrotoxicity

## Abstract

Nephrotoxic medication exposure is nearly ubiquitous in hospitalized patients and represents one of the most common causes of acute kidney injury (AKI) in the hospitalized setting. Although provision of medications that are nephrotoxic has led to improved outcomes in terms of treatment of underlying illness, unnecessary nephrotoxic medication exposure can be viewed as a potentially modifiable adverse safety event if AKI can be prevented. The advancements in electronic health record development, standardization of AKI definitions, and the ability to identify AKI risk and development in near real time provide opportunities to reduce harm from nephrotoxicity.

## Introduction

Many of the advancements in the field of medicine have been achieved by the development of medications targeted at severe acute and chronic underlying conditions such as infection, heart disease, and cancer. Unfortunately, many of these medications are nephrotoxic, meaning that they are injurious to the kidney and can cause acute and, in some cases, irreparable damage to the kidneys. Nephrotoxic medication exposure is nearly ubiquitous for hospitalized patients and is one of the most common causes of acute kidney injury (AKI) in the hospital ^1, 2^.

There is the potential perception that provision of nephrotoxic medications and the associated AKI are “just the costs of doing business” and a necessary evil of tertiary health care. Such a perception ignores an opportunity to expose patients to only the nephrotoxic medications they need for the time that they need them. Furthermore, since many nephrotoxic medications are excreted by the kidneys, they or their metabolites can accumulate and cause worsening AKI or other systemic organ injury and dysfunction.

The confluence of standardized and validated serum creatinine-based AKI diagnostic and severity criteria and the requirement for, and adoption of, electronic health records provides an opportunity to expeditiously catalog nephrotoxic medication exposure and burden as well as AKI development and severity. This article will highlight recent efforts to reduce unnecessary harm from indiscriminate nephrotoxic medication exposure.

## Nephrotoxic medications and acute kidney injury: the perfect combination for electronic health records surveillance

AKI is currently defined as an abrupt rise in serum creatinine or decrease in urine output ^3^. The adoption of standardized AKI criteria clearly demonstrates an independent association among AKI development, severity, and morbidity in hospitalized patients ^4– 6^. Hospital-wide AKI detection systems, or “sniffers”, demonstrate very low false-positive and false-negative rates (1.7% and 0.2%, respectively) ^7^, AKI rates of greater than 10%, and greater ascertainment of AKI than reliance on administrative coding ^8, 9^. We have found that systematic surveillance for AKI yields higher AKI detection rates than does reliance on ICD-9 (International Classification of Diseases, Ninth Revision) coding for hospitalized children exposed to nephrotoxic medications ^10^.

Systematic surveillance and detection of AKI have the ability to improve patient outcomes. When real-time AKI alerts were sent to intensive care unit physicians combined with targeted AKI interventions, including fluid bolus, initiation of vasopressor medications, and prescription of diuretics ^11^, the rate of interventions was significantly increased and time to interventions was decreased in the alert period, and patients in the alert group were more likely to demonstrate a return to baseline kidney function within 8 hours of AKI detection. To address nephrotoxic medication-associated AKI directly, McCoy *et al*. created an electronic health record integrated alert for patients who had an active recurring order for nephrotoxic medications or medications with renal excretion and who developed AKI ^12^. Their alert presented the providers with a visual cue of the medication of interest and the potential options to either adjust the dose or discontinue or continue the medication(s). They observed improvements in time with nephrotoxic medication dose adjustment or avoidance.

However, these alerts highlight a reactive posture toward AKI epidemiology, waiting for AKI to occur. Since the electronic health record contains data tables for medications prescribed and delivered to a patient, the opportunity exists to identify patients at risk for nephrotoxic medication-associated AKI. It is well known that certain combinations of medications, including angiotensin-converting enzyme inhibitors/angiotensin receptor blockers plus diuretics plus non-steroidal anti-inflammatory drugs (NSAIDs), known as the “triple whammy”, are associated with increased rates of AKI ^13, 14^. Furthermore, a combination of two of these classes of medications was associated with an increased risk of AKI. Thus, in the setting of AKI and in patients at risk for AKI, such as those with dehydration or acute infection, clinicians must balance the risk of nephrotoxicity versus the therapeutic benefit that led to their prescription in the first place.

The pediatric population provides a less confounded group for nephrotoxic medication-associated AKI assessment. In non-critically ill children, the rate of AKI doubled in children who received three or more nephrotoxic medications versus two or fewer ^2^. This observation, in addition to a 19 **–**31% AKI rate observed in children with prolonged aminoglycoside exposure ^15^, led to the development of a prospective AKI monitoring program called Nephrotoxic Injury Negated by Just-in-time Action (NINJA) ^16, 17^. NINJA uses an automated program to extract data in near real time from electronic health records to flag non-critically ill hospitalized children exposed to three or more nephrotoxic medications simultaneously or an intravenous aminoglycoside for 3 or more consecutive days. Exposed patients underwent systematic surveillance with a daily serum creatinine measurement. In the first year, NINJA revealed that 25% of exposed patients developed AKI and that more than 50% experienced severe AKI, defined as a doubling of serum creatinine from baseline. In the first 3 years of the NINJA project at our center, we observed a 38% decrease in the rate of three nephrotoxic medication exposures and a concomitant 64% decrease in AKI rates, which was associated with more than 600 exposures and nearly 400 AKI episodes avoided ^18^. Although the NINJA is a surveillance program for non-critically ill hospitalized children, it is not unreasonable to expect that the contribution of nephrotoxic medications to AKI acquired in the intensive care unit would likely be lessened as well with systematic surveillance of nephrotoxic medication burden.

Single-drug nephrotoxicity may also occur at higher rates than previously recognized. For example, Misurac *et al*. assessed the rate of AKI in hospitalized children receiving NSAIDs at a single center ^19^. The authors found that 6.6% of children receiving NSAIDs developed AKI, even though 75% received doses in the recommended range.

## What can be done?

Given the reliance on nephrotoxic medications to treat many critical illnesses, there may be an understandable reluctance to modify their provision. And once a patient develops AKI from nephrotoxic medication or other causes, there is little that can be done to alter the course of AKI, as the damage has already occurred. However, diligence with respect to review of nephrotoxic medication burden, altered excretion in patients with AKI, and searching for equally efficacious but less nephrotoxic medications should be undertaken daily. As noted above, the triple whammy reported in adults and pediatric data demonstrating increased AKI rates when a third nephrotoxic medication is prescribed should prompt clinicians and pharmacists to be deliberate when adding a third nephrotoxic medication.

## What does the future hold?

Two potential strategies currently under investigation aim to optimize risk assessment for nephrotoxic medication-associated AKI: (1) novel kidney injury biomarker assessment and (2) assessment of genetic predisposition to specific nephrotoxic medication-associated AKI. Whereas much of the biomarker work has focused on detecting subclinical kidney injury with known nephrotoxins ^20^, a recent study demonstrated that urinary neutrophil gelatinase-associated lipocalin (NGAL) was associated with decreased tobramycin clearance and an increased area under the concentration-time curve in patients with cystic fibrosis ^21^. Because NGAL and tobramycin use a similar transport receptor (megalin), these results suggest that other biomarkers may also be predictive of disordered pharmacokinetics for other medications depending on their respective transport physiologies. Finally, a recent combined adult and pediatric international multicenter initiative has been aimed at assessing genetic predisposition to medication-associated AKI by using genome-wide association studies ^22^.

## Abbreviations

AKI, acute kidney injury; NGAL, neutrophil gelatinase-associated lipocalin; NINJA, Nephrotoxic Injury Negated by Just-in-time Action; NSAID, non-steroidal anti-inflammatory drug.
